# Associations of dietary sources of antioxidant intake and NAFLD: NHANES 2017–2020 and Mendelian randomization

**DOI:** 10.3389/fnut.2024.1447524

**Published:** 2024-10-30

**Authors:** Zilong Yue, Ziming Jiang, Long Qian, Lele Li, Xianliang Qi, Kaifeng Hu

**Affiliations:** ^1^Department of Gastrointestinal Surgery, The First Affiliated Yijishan Hospital of Wannan Medical College, Wuhu, Anhui, China; ^2^General Surgery Department, Guoyang Branch of Anhui Provincial Hospital, Bozhou, Anhui, China; ^3^Department of Urology, Shanghai 10th People’s Hospital Affiliated to Tongji University, Shanghai, China; ^4^General Surgery Department, Wuhu Hospital of Traditional Chinese Medicine, Wuhu, Anhui, China

**Keywords:** dietary sources of antioxidant, non-alcoholic fatty liver disease, NHANES, vitamin E, Mendelian randomization

## Abstract

**Purpose:**

To determine the association between dietary antioxidant sources and non-alcoholic fatty liver disease (NAFLD).

**Methods:**

In this observational study, we utilized NHANES 2017–2020 data to identify the factors associated with NAFLD in dietary antioxidant sources via weighted multivariate logistic regression models. Then, Mendelian randomization (MR) was applied to investigate the effect of dietary antioxidant sources on NAFLD at the genetic level.

**Results:**

Of the six dietary sources of antioxidants, only vitamin E (Vit E) was significantly associated with NAFLD (OR = 0.98; 95% CI: 0.97–0.99; *p* = 0.001). Upon adjusting for all covariates, it was determined that the highest quartile of dietary Vit E intake was associated with a decreased NAFLD occurrence compared with the lowest quartile of dietary Vit E intake (*p* < 0.001). The results of IVW-MR analysis revealed an association between Vit E and NAFLD (OR = 0.028; *p* = 0.039).

**Conclusion:**

Our research indicates a negative and linear relationship between daily vitamin E intake and NAFLD.

## Introduction

Non-alcoholic fatty liver disease (NAFLD) is one of the most common chronic liver conditions worldwide. Studies suggest that the prevalence of NAFLD is more than 30% among adults in the United States and China (1, 2). Based on its histological type, NAFLD can be classified into two types: non-alcoholic fatty liver (NAFL) and non-alcoholic steatohepatitis (NASH), and NASH primarily arises from the advancement of NAFL (3, 4). At present, end-stage liver disease triggered by NAFLD is the primary reason for liver transplantation, accounting for 8.4% of liver transplant cases in Europe and 17.38% in the United States (5, 6). This has been showing an increasing trend each year. NAFLD imposes a significant strain on global public health. Therefore, determining ways to effectively prevent or ameliorate NAFLD is urgently warranted.

Options for the pharmacological treatment of NAFLD are limited; however, evidence suggests the effectiveness of resmetirom in treating NASH (7, 8). Nevertheless, it has not yet received clinical approval. Therefore, the current treatment for NAFLD mainly involves lifestyle intervention and management of comorbidities. Oxidative stress is defined as an imbalance between oxidant production in the body and cellular antioxidant defenses; this ultimately leads to cellular dysfunction and tissue damage, and is an important mechanism in the development of NAFLD (9–11). Oxidative stress can cause increased expression of IRE1a and ATF4, which leads to increased autophagy and activation of hepatic stellate cells (12). The exogenous antioxidants can modulate the damage caused by oxidative stress by neutralising reactive oxygen species (13). Many studies on mice have revealed that NAFLD incidence can be decreased by controlling oxidative stress (14–16). So, we conjecture that there is a possible correlation between the intake of exogenous antioxidants in the daily diet and the onset of NAFLD. Vitamin A (Vit A), vitamin C (Vit C), vitamin E (Vit E), carotenoids, selenium and zinc are common exogenous antioxidants found in our daily diet. Dietary antioxidant sources intervene in hepatic metabolism through multiple metabolic pathways. Vit A can inhibit fatty acid oxidation in the liver (17). Xie ZQ’s study showed that increased serum levels of Vit C reduced the risk of NAFLD (18). Furthermore, Panera et al. have reported that exogenous Vit E supplementation can ameliorate NAFLD-induced liver fibrosis (19). Reduced serum zinc levels can lead to increased hepatic fibrosis in NAFLD patients (20). Dietary selenium consumption can reduce oxidative stress in hepatocytes by enhancing the synthesis of selenoprotein P1 (21). These evidence suggest that there may be some association between antioxidants and NAFLD. After consulting relevant literature, we discovered a lack of studies investigating the relationship between dietary intake of exogenous antioxidants and NAFLD.

In this study, we plan to identify exogenous antioxidants in the daily diet related to NAFLD. Then further analyze the relationship between these selected antioxidants and NAFLD.

## Methods

### Study population

All data were derived from The National Health and Nutrition Examination Survey (NHANES). The ethics review board of the National Center for Health Statistics approved the NHANES survey protocol, with all participants providing written informed consent before the survey. We selected participants between 2017 and 2020. The exclusion criteria were as follows: (1) age < 20 years, (2) absence of complete dietary data, and (3) incomplete follow-up data.

### Exposure and outcomes

Two nonconsecutive 24 h dietary recall interviews were conducted to collect information on the intake of NHANES dietary sources of exogenous antioxidants. Subsequently, the data were converted into estimates of nutrient intake via the Food and Nutrient Database for Dietary Studies of the United States Department of Agriculture. Vit A, Vit C, Vit E, carotenoids, selenium, and zinc are the primary exogenous antioxidants ingested in the daily diet. Based on the NHANES questionnaire results, we determined the intake of dietary sources for each participant.

To further investigate the overall effect of antioxidants from multiple dietary sources, the composite dietary antioxidant index (CDAI) for each participant in NHANES was calculated based on their dietary records. The CDAI represents a composite score of the above six dietary antioxidant intakes. CDAI calculations follow a previously established and validated methodology (22), as follows:


$$\mathrm{CADI}=\overset{6}{\underset{i=1}{\sum }}\frac{x_{i}-u_{i}}{S_{i}}$$

$x_{i}$ is the daily intake of each antioxidant, $u_{i}$ is the mean value of $x_{i}$ , and $S_{i}$ is the standard deviation of $u_{i}$ (23). NAFLD was defined in this study as a Controlled Attenuation Parameter (CAP) score of ≥248 dB/m in the absence of alcoholism and liver disease (24). Participants with NAFLD were defined as NAFLD group and patients without NAFLD were defined as No NAFLD group.

### Covariates

Based on the participant-related data contained in the NHANES database, we selected the following variables as covariates: sex, age, education, BMI, smoking history, diabetes history, fasting blood glucose, red blood cell count (RBC), hemoglobin A1C (HbA1C), lymphocyte count (LN), neutrophil count (NEUT), platelet count (PLT), hemoglobin (Hb), white blood cell count (WBC), C-reactive protein (CRP), Alaninetransaminase (ALT), Alaninetransaminase (AST), and blood lipids.

### Mendelian randomization

**Figure 1:**
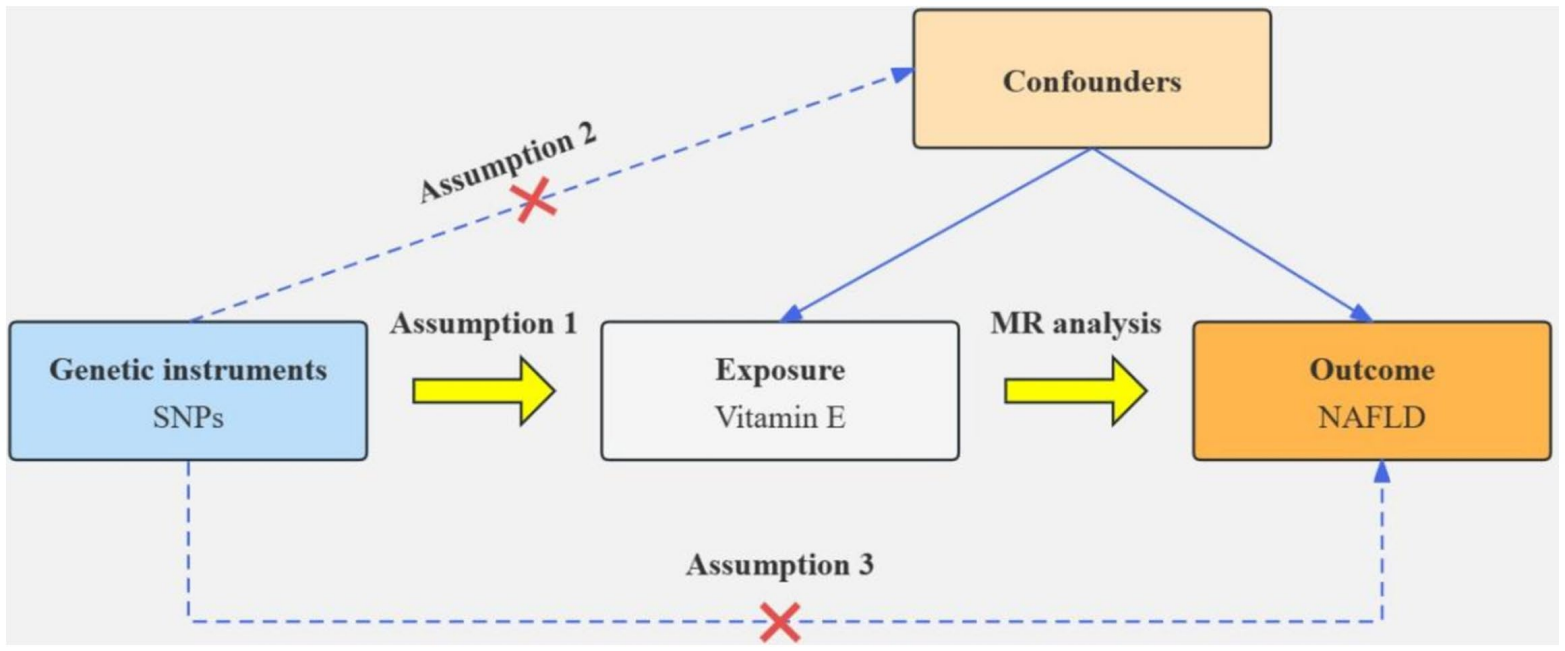
Mendelian randomization flow chart.

Based on the results of the cross-sectional study, antioxidants associated with NAFLD were selected as the exposure factor. Single nucleotide polymorphisms (SNPs) closely associated with specific exogenous antioxidants and with correlations satisfying *p* < 5 × 10^−5^ were selected as instrumental variables (IVs). We used *r*^2^ < 0.001 and kb >10,000 as linkage disequilibrium thresholds to ensure the independence of IV (25). To quantify the strength of the genetic tool, SNP with F-statistic less than 10 are excluded. Inverse variance weighted (IVW) was used as the primary method for MR analysis. Furthermore, MR-Egger regression, weighted median, and other IVW-complementing methods were used. Cochran’s Q test was used to assess heterogeneity among SNPs, whereas MR-Egger regression intercepts were used to assess horizontal pleiotropy. A leave-one-out analysis was performed to estimate the potential effect of individual SNPs on the causal relationship between NAFLD and associated antioxidant intake. Figure 1 illustrates the flow chart of MR analysis.

### Statistical analysis

R (4.2.2) was used to perform statistical analysis. A *p*-value of <0.05 was considered a statistically significant difference. Continuous variables were expressed using mean (standard deviation), whereas categorical variables were expressed using *n* (%). For continuous variables, group comparisons were conducted using the student *t*-test. In contrast, the chi-square test was used for categorical variables. Logistic regression models were utilized to analyze the relationship between exogenous antioxidants and NAFLD. The covariates in the crude model were not adjusted, and model 1 comprised all covariates. Restricted cubic splines analysis was used to determine if a non-linear relationship exists between antioxidant intake and NAFLD.

## Results

### Baseline characteristics

**Figure 2:**
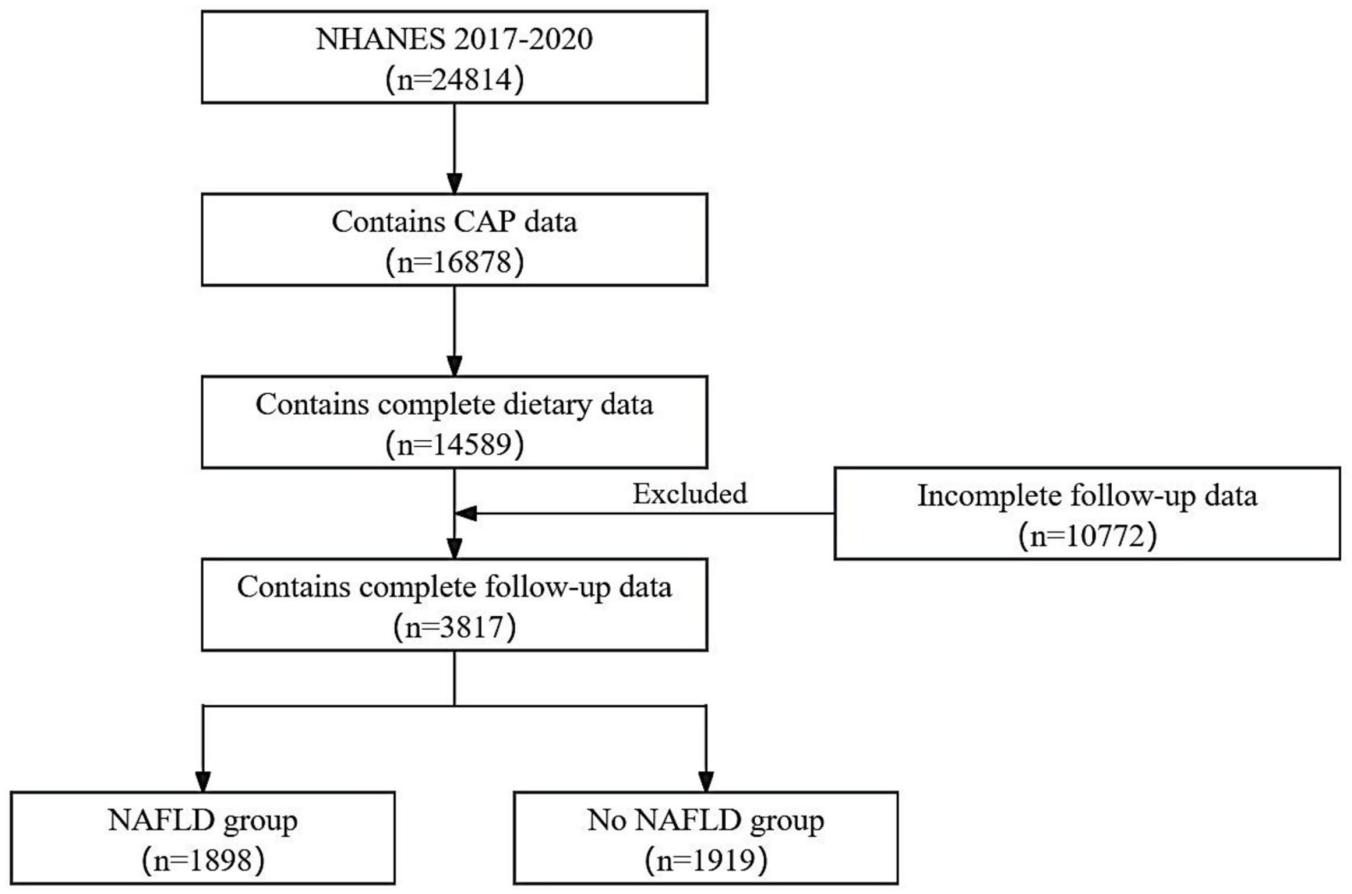
The participant selection flow chart.

This study included 3,817 respondents, of which 1926 (50.5%) were males and 1891 (49.5%) were females. Figure 2 illustrates the detailed flowchart. Table 1 presents the baseline demographic characteristics. The NAFLD and the No NAFLD groups exhibited significant differences in age, race, education level, body mass index, hypertension prevalence, and diabetes prevalence (*p* < 0.05 for all). Patients with NAFLD exhibited higher age, education level, and prevalence of diabetes and hypertension.

**Table 1:** Baseline demographic characteristics.

Variables	Total (*n* = 3,817)	NAFLD (*n* = 1898)	No NAFLD (*n* = 1919)	*p*
**Age, years**	51.0 ± 16.8	52.1 ± 16.2	49.9 ± 17.3	<0.001
**Gender, *n* (%)**				0.202
Female	1891 (49.5)	960 (50.6)	931 (48.5)	
Male	1926 (50.5)	938 (49.4)	988 (51.5)	
**Race/Ethnicity, *n* (%)**				<0.001
Mexican American	641 (16.8)	318 (16.8)	323 (16.8)	
Other Hispanic	469 (12.3)	192 (10.1)	277 (14.4)	
Non-Hispanic White	1,370 (35.9)	695 (36.6)	675 (35.2)	
Non-Hispanic Black	759 (19.9)	384 (20.2)	375 (19.5)	
Other race	989 (25.9)	483 (25.4)	506 (26.4)	
**Education level, *n* (%)**				<0.001
<9 years	352 (9.2)	140 (7.4)	212 (11)	
9–11 years	406 (10.6)	192 (10.1)	214 (11.2)	
Senior high schools or GED	871 (22.8)	437 (23)	434 (22.6)	
Some college or associate degree	1,199 (31.4)	646 (34)	553 (28.8)	
Colleges or above	989 (25.9)	483 (25.4)	506 (26.4)	
**BMI, *n* (%)**				<0.001
<25 Kg/m^2^	733 (19.2)	223 (11.7)	510 (26.6)	
≥25 Kg/m^2^	3,084 (80.8)	1,675 (88.3)	1,409 (73.4)	
**Smoke, *n* (%)**				0.15
Yes	1,631 (42.7)	789 (41.6)	842 (43.9)	
No	2,186 (57.3)	1,109 (58.4)	1,077 (56.1)	
**Hypertension, *n* (%)**				<0.001
Yes	1,528 (40.0)	829 (43.7)	699 (36.4)	
No	2,289 (60.0)	1,069 (56.3)	1,220 (63.6)	
**Diabetes, *n* (%)**				<0.001
Yes	685 (17.9)	389 (20.5)	296 (15.4)	
No	3,132 (82.1)	1,509 (79.5)	1,623 (84.6)	
**WBC, 1000 cells/uL**	6.9 ± 2.7	6.9 ± 2.0	6.9 ± 3.2	0.738
**LN, 1000 cells/uL**	2.1 ± 1.9	2.1 ± 0.7	2.1 ± 2.6	0.504
**NEUT, 1000 cells/uL**	4.0 ± 1.6	4.0 ± 1.6	4.0 ± 1.5	0.238
**RBC, 1000 cells/uL**	4.8 ± 0.5	4.8 ± 0.5	4.8 ± 0.5	0.039
**Hb, g/dL**	14.2 ± 1.5	14.2 ± 1.5	14.1 ± 1.5	0.002
**PLT, 1000 cells/uL**	238.7 ± 61.8	243.5 ± 62.0	234.0 ± 61.2	<0.001
**CRP, mg/L**	4.4 ± 8.0	4.8 ± 8.1	4.1 ± 7.9	0.005
**ALT, U/L**	24.9 ± 15.8	24.7 ± 15.8	25.1 ± 15.8	0.372
**AST, U/L**	23.9 ± 18.0	22.2 ± 12.8	25.6 ± 21.9	<0.001
**Glu, mmol/L**	6.4 ± 2.3	6.6 ± 2.3	6.3 ± 2.3	<0.001
**HbA1c, %**	5.9 ± 1.3	6.0 ± 1.3	5.9 ± 1.3	<0.001
**TC, mg/dL**	4.9 ± 1.1	4.9 ± 1.1	4.9 ± 1.1	0.087
**HDL, mg/dL**	1.4 ± 0.4	1.3 ± 0.4	1.4 ± 0.5	<0.001
**TG, mg/dL**	1.3 ± 0.8	1.4 ± 0.8	1.2 ± 0.7	<0.001
**LDL, mg/dL**	2.9 ± 0.9	2.9 ± 0.9	2.9 ± 0.9	0.692
**Vit A, μg**	584.9 ± 562.9	589.2 ± 527.7	580.7 ± 595.8	0.639
**Vit C, mg**	78.7 ± 91.6	80.4 ± 96.8	77.0 ± 86.1	0.249
**Vit E, mg**	8.8 ± 6.1	9.1 ± 6.3	8.5 ± 5.8	<0.001
**Se, μg**	114.7 ± 68.1	113.7 ± 70.6	115.6 ± 65.5	0.377
**Zn, mg**	10.8 ± 12.2	10.9 ± 16.2	10.6 ± 6.0	0.471
**Carotenoid, μg**	2267.5 ± 4282.5	2334.3 ± 4343.5	2201.6 ± 4221.4	0.339

WBC, white blood cell count; LN, lymphocyte count; NEUT, neutrophil count; RBC, red blood cell count; Hb, hemoglobin; PLT, platelet count; CRP, C-reactive protein; ALT, alaninetransaminase; AST, alaninetransaminase; Glu, fasting blood glucose; HbA1c, hemoglobin A1C; TC, total cholesterol; HDL, high density lipoprotein; TG, triglyceride; LDL, low density lipoprotein; Vit A, vitamin A; Vit C, vitamin C; Vit E, vitamin E.

### Association of exogenous antioxidants and NAFLD

Table 2 summarizes the results of logistic regression analysis. Among the six exogenous antioxidants included in this study, only Vit E was noted to be associated with NAFLD occurrence (OR = 0.98; 95% CI: 0.97–0.99; *p* = 0.001).

**Table 2:** Association of dietary antioxidants and NAFLD.

Dietary antioxidants	OR (95% CI)	*p*
Vitamin A	1.00 (1.00 ~ 1.00)	0.639
Vitamin C	1.00 (1.00 ~ 1.00)	0.250
Vitamin E	0.98 (0.97 ~ 0.99)	0.001
Selenium	1.00 (1.00 ~ 1.00)	0.378
Zinc	1.00 (0.99 ~ 1.00)	0.482
Carotenoid	1.00 (1.00 ~ 1.00)	0.339

OR, odds ratio; CI, confidence interval.

### Association between Vit E and NAFLD

**Figure 3:**
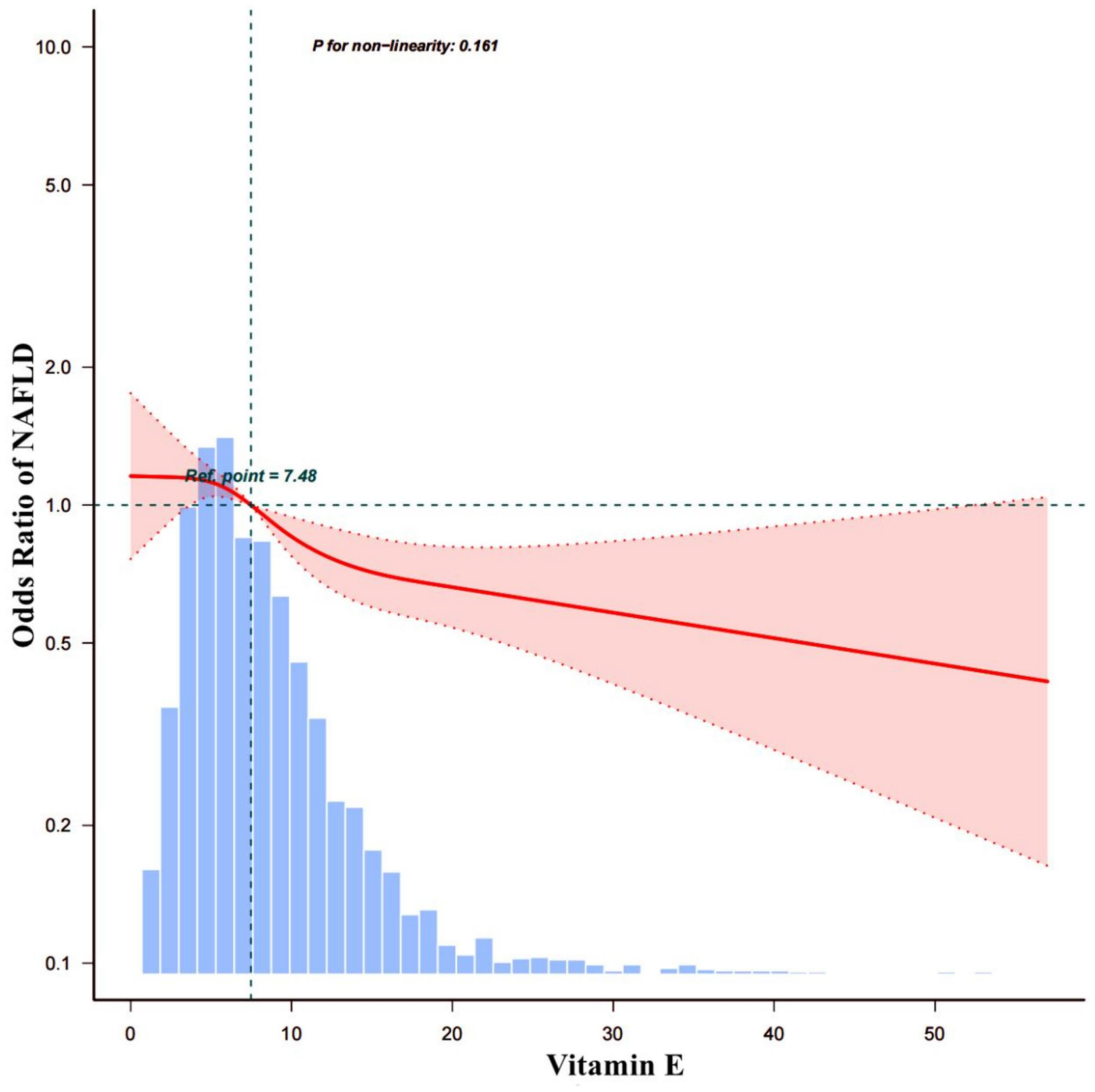
The dose–response relationship between vitamin E and the prevalence of NAFLD.

To investigate the correlation between Vit E and NAFLD, two weighted models were constructed. The results are summarized in Table 3. The crude model’s findings showed that a daily intake of Vit E in the Q4 range reduced the likelihood of developing NAFLD by 26% (OR = 0.74; 95% CI: 0.62–0.89; *p* = 0.001). After we adjusting for all covariates in model 1, we noted that Vit E intake in Q3 (OR = 0.78; 95% CI: 0.63–0.96; *p* = 0.019) and Q4 (OR = 0.63; 95% CI: 0.5–0.79; *p* < 0.001) was significantly associated with a reduced risk of NAFLD. Using a restricted cubic spline plot, we further analyzed the relationship between Vit E intake and NAFLD and noted a linear relationship (*p* = 0.124; Figure 3).

**Table 3:** Association between Vit E and NAFLD.

Characteristics	Crude model		Model 1	
	OR (95% CI)	*p*	Adj. OR (95% CI)	*p*
**Vit E**	0.98 (0.97 ~ 0.99)	0.001	0.97 (0.96 ~ 0.98)	<0.001
Categorical Vit E				
Q 1	Ref		Ref	
Q 2	1.04 (0.87 ~ 1.24)	0.7	1.01 (0.83 ~ 1.23)	0.89
Q 3	0.84 (0.7 ~ 1.01)	0.063	0.78 (0.63 ~ 0.96)	0.019
Q 4	0.74 (0.62 ~ 0.89)	0.001	0.63 (0.5 ~ 0.79)	<0.001

Ref, reference. Crude model: none variables were adjusted. Model 1: adjusted for all variables.

### Subgroup analysis of the association between Vit E and NAFLD

In males, Q3 doses of Vit E reduced NAFLD incidence by 32% (OR = 0.68; 95% CI: 0.52–0.89; *p* = 0.006), and Q4 doses by 41% (OR = 0.59; 95% CI: 0.46–0.77; *p* < 0.001). After adjusting for covariates, these effects were amplified. However, no significant reduction was observed in females.

**Figure 4:**
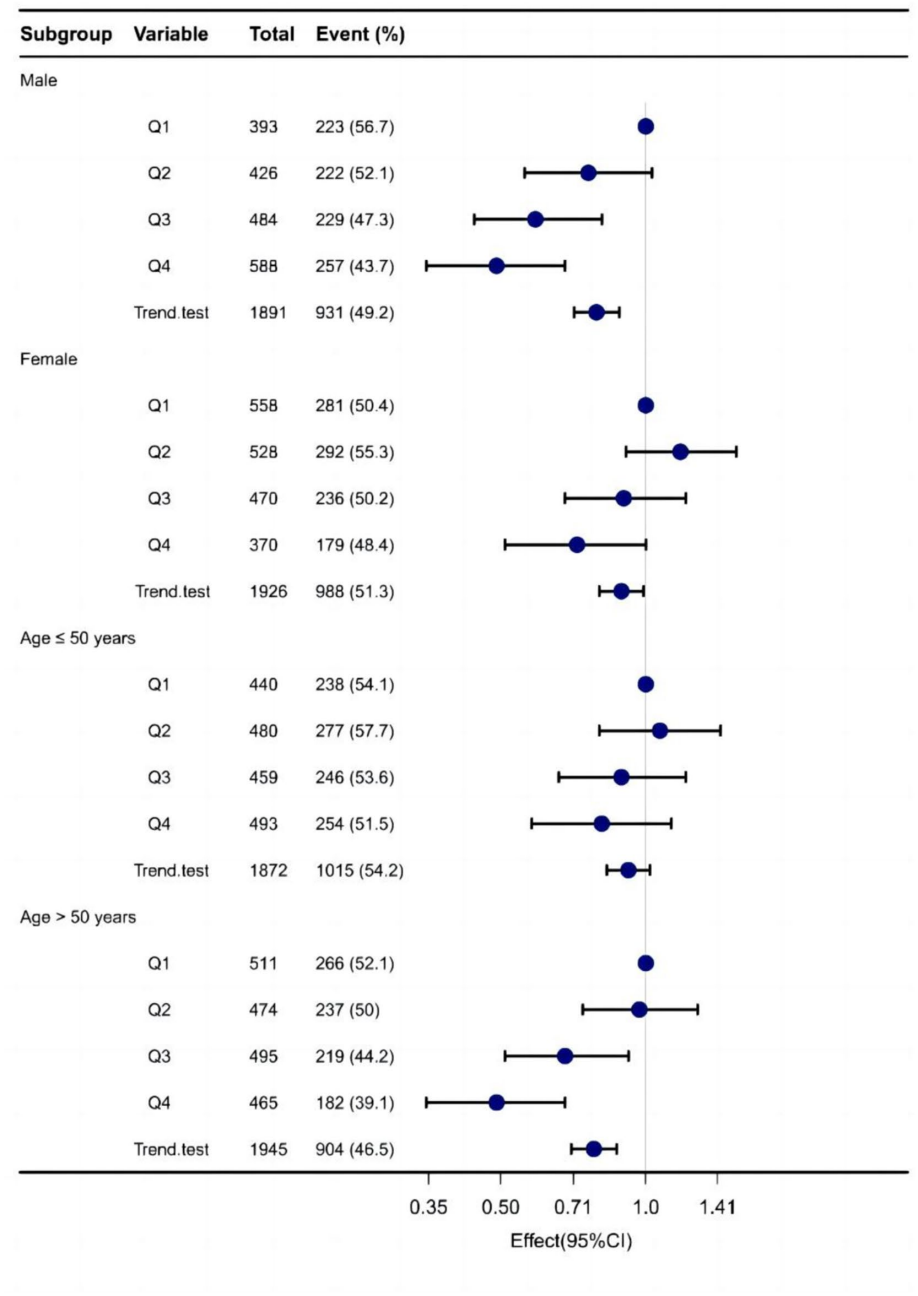
Forest plot demonstrates the risk association between different doses of dietary sources of vitamin E and NAFLD across various subgroups.

For participants over 50 years, Q3 (OR = 0.73; 95% CI: 0.57–0.94; *p* = 0.013) and Q4 (OR = 0.59; 95% CI: 0.46–0.76; *p* < 0.001) doses significantly reduced NAFLD risk, even after covariate adjustment. No reduction was found in those under 50 years. Table 4 and Figure 4 summarize the results.

**Table 4:** Subgroup analysis for the association between Vit E and NAFLD.

Characteristics	Crude model		Model 1	
	OR (95% CI)	*p*	Adj. OR (95% CI)	*p*
Male				
Q 1	Ref		Ref	
Q 2	0.83 (0.63 ~ 1.09)	0.184	0.76 (0.56 ~ 1.03)	0.078
Q 3	0.68 (0.52 ~ 0.89)	0.006	0.59 (0.44 ~ 0.81)	0.001
Q 4	0.59 (0.46 ~ 0.77)	<0.001	0.49 (0.71 ~ 0.88)	<0.001
P for trend	<0.001		<0.001	
Female				
Q 1	Ref		Ref	
Q 2	1.22 (0.96 ~ 1.55)	0.103	1.18 (0.91 ~ 1.54)	0.215
Q 3	0.99 (0.78 ~ 1.27)	0.963	0.90 (0.68 ~ 1.21)	0.495
Q 4	0.92 (0.71 ~ 1.20)	0.555	0.72 (0.51 ~ 1.00)	0.050
P for trend	0.369		0.031	
Age ≤ 50 years				
Q 1	Ref		Ref	
Q 2	1.16 (0.89 ~ 1.50)	0.270	1.07 (0.80 ~ 1.43)	0.650
Q 3	0.98 (0.75 ~ 1.27)	0.881	0.89 (0.66 ~ 1.21)	0.462
Q 4	0.90 (0.70 ~ 1.17)	0.433	0.81 (0.58 ~ 1.13)	0.207
P for trend	0.230		0.126	
Age > 50 years				
Q 1	Ref		Ref	
Q 2	0.92 (0.72 ~ 1.18)	0.519	0.97 (0.74 ~ 1.28)	0.839
Q 3	0.73 (0.57 ~ 0.94)	0.013	0.68 (0.51 ~ 0.92)	0.011
Q 4	0.59 (0.46 ~ 0.76)	<0.001	0.49 (0.35 ~ 0.68)	<0.001
P for trend	<0.001		<0.001	

Ref, reference. Crude model adjusted for: none. Model 1 adjusted for: all variables.

### MR analysis

**Figure 5:**
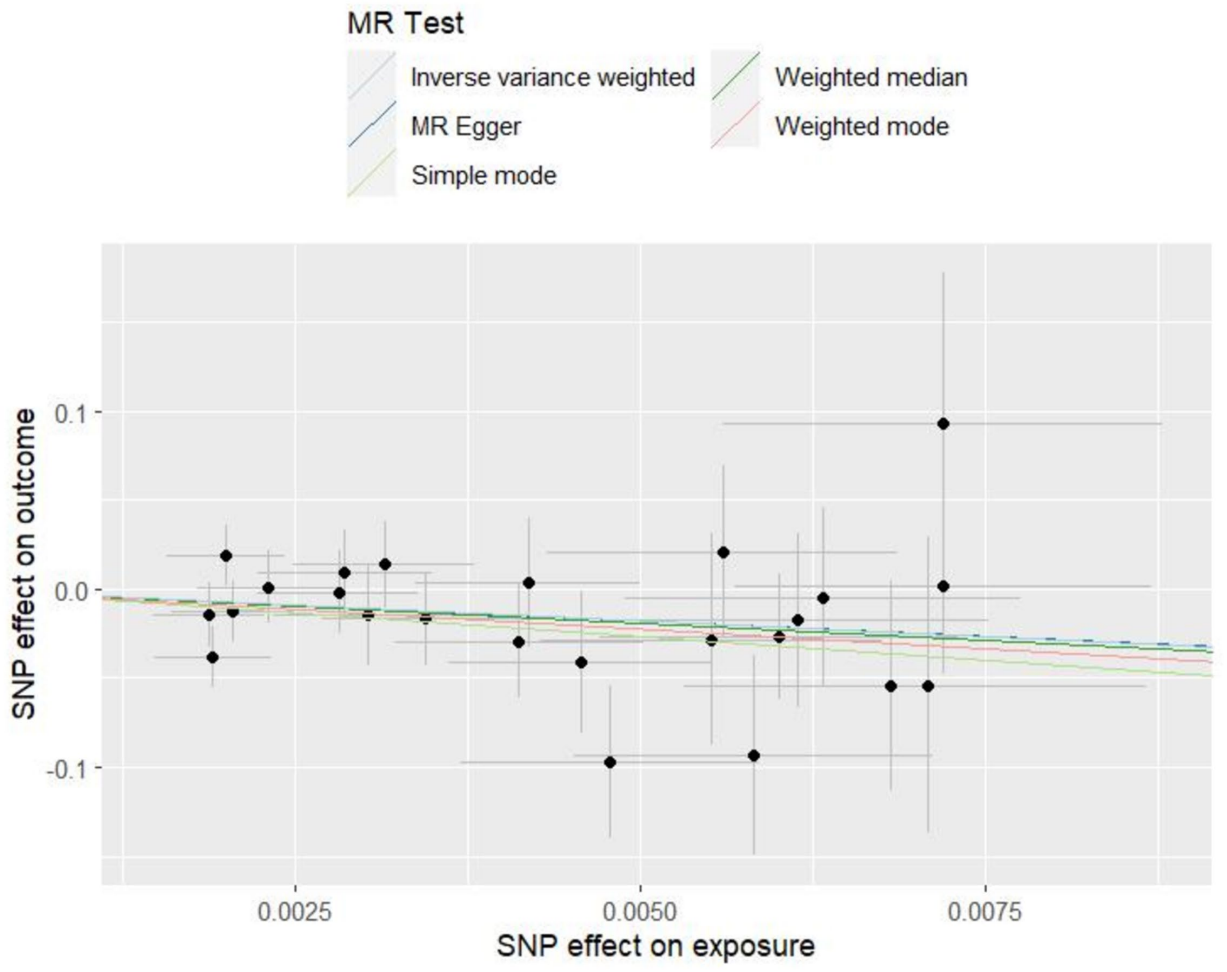
Scatter plots of Mendelian randomization tests assessing the effect of dietary sources of vitamin E on NAFLD.

**Figure 6:**
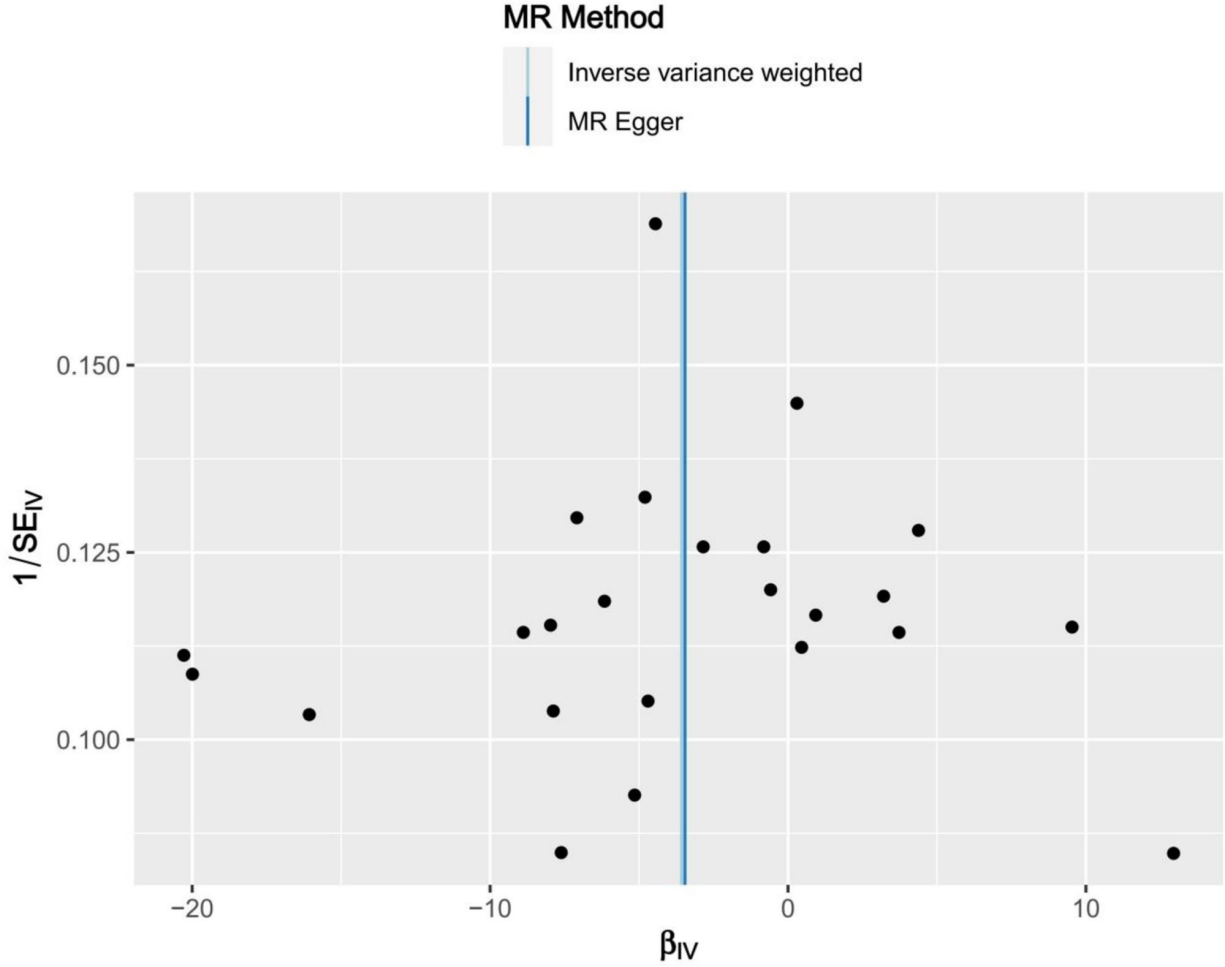
Funnel plots of Mendelian randomization tests assessing the effect of dietary sources of vitamin E on NAFLD.

Genome-wide association study (GWAS) statistics for NAFLD were obtained from a genome-wide meta-analysis predominantly comprising European populations, which was conducted by Ghodsian et al. (26). GWAS data for Vit E supplementation were collected from UK Biobank: ukb-a-463. Based on the selection criteria for SNPs, 24 SNPs were ultimately included to investigate the association between Vit E intake and NAFLD (Supplementary Table S1). IVW analysis revealed a causal connection between Vit E supplementation and NAFLD risk (OR = 0.028; *p* = 0.039). Analysis of scatter and funnel plots suggested a relatively balanced sample selection, with no evident bias (Figures 5, 6). IVW and MR-Egger regression analyses indicated that Cochran’s Q statistic was 17.313 (*p* = 0.794) and 17.312 (*p* = 0.746), respectively. This suggests the absence of heterogeneity among SNPs. Leave-one-out analysis revealed that upon removing any one SNP, the results of the remaining SNPs consistently fell on the same side of the invalid line. This verifies the robustness of the results of the MR analysis (Supplementary Figure S1).

## Discussion

Studies have confirmed a close association between exogenous antioxidants and the occurrence and development of various diseases (27, 28). However, studies on the association between consuming exogenous antioxidants from dietary sources and NAFLD are limited. NAFLD progression is intricately associated with dietary patterns (29). In the absence of effective pharmacological interventions for NAFLD, we aimed to explore potential treatment modalities for NAFLD by improving lifestyle habits and dietary changes, among other factors.

Our study is the first attempt to utilize NHANES data to investigate the correlation between exogenous antioxidant intake and NAFLD and to establish the association between them at the genetic level via MR analysis. By analyzing exogenous antioxidants from the six common dietary sources included in this study, we discovered that only Vit E intake is significantly associated with the risk of developing NAFLD (*p* < 0.05). Vit E, an essential nutrient for the human body, comprises the benzodihydropyran structure and exhibits *α*-tocopherol bioactivity. It belongs to a class of substances primarily found in various plants (30). In addition, Vit E acts as a chain-breaking antioxidant, combating free radicals and scavenging them (31).

Oxidative stress may fundamentally affect NAFLD progression, with a strong correlation between excessive reactive oxygen species generation and NAFLD-associated hepatocyte death (32). Moreover, oxidative stress can increase the levels of IL-2, TNF, and IL-2, which may contribute to hepatic fibrosis (33, 34). Exogenous Vit E supplementation can inhibit JNK-mediated inflammatory signaling pathways to attenuate inflammation levels in patients with NAFLD (35). Vit E supplementation, as demonstrated in a study by Bai Y et al., was found to activate the AMPK pathway and reduce oxidative stress, resulting in improved NAFLD in rats (36).

Several studies have indicated a close relationship between Vit E and NAFLD. To determine into the correlation between the intake of various amounts of Vit E and NAFLD, we developed two distinct models. Both models revealed that the risk of NAFLD was significantly decreased (*p* < 0.05) with Vit E intake up to Q3 and Q4. Sanyal AJ et al. have suggested that the daily supplementation of 800 IU of Vit E can be used to treat NAFLD (37). Therefore, increasing exogenous Vit E intake may be an effective measure to prevent NAFLD development.

Subgroup analyses revealed a significant association between Vit E and NAFLD in men and those older than 50 years (*p* < 0.05), but not in women and those younger than 50 years (*p* > 0.05). Studies have revealed that the dietary patterns of men are dominated by “convenience, red meat, and alcohol,” which often results in oxidative stress and chronic inflammation in the body (38–41). At the same time women produce higher levels of oestrogen which can act as an antioxidant (42). Therefore, in male patients, supplementing Vit E is more essential to decrease oxidative stress in the body and subsequently decrease NAFLD risk. Vit E can efficiently enhance age-related disruption of immune and inflammatory reactions (43). In the present study, we suggested that Vit E can effectively decrease NAFLD risk in the elderly population. Hemilä H’s findings showed that a daily intake of 50 mg of vitamin E reduced the risk of pneumonia by 67% in older people, but did not have the same effect in younger people (44). Study has shown that aging causes elevated levels of reactive oxygen species in the body (45), which promotes oxidative stress. Therefore, age can serve as a determining factor in assessing the need for Vit E, and additional studies are warranted to substantiate this. Although studies on using Vit E for treating NAFLD are available, critical evidence to support the use of Vit E as a key measure for treating and preventing NAFLD is lacking.

Observational studies are often affected by confounding factors; however, MR can analyze the relationship between exposure factors and outcomes while decreasing the effect of confounding factors (46). Based on the conclusions drawn from the cross-sectional study, we further validated the relationship between Vit E intake and NAFLD using MR analysis. IVW-MR analysis revealed an association between Vit E intake and NAFLD. Furthermore, it revealed that Vit E intake decreases NAFLD risk. The results of observational studies and MR analyses support the idea that Vit E supplementation decreases NAFLD risk. Combined with the findings of other related studies (47–49), we hypothesize that Vit E intervenes in NAFLD development primarily by regulating oxidative stress, inflammatory responses, gene expression, and cell signaling. However, the specific molecular mechanism underlying the role of Vit E in NAFLD remains unclear. Therefore, additional experiments are warranted to verify this.

In our study, we analyzed the common exogenous antioxidants in the daily diet and finally observed that Vit E can effectively decrease NAFLD risk. This provides some reference for treating and preventing NAFLD in clinical settings. Our study is the first to use a cross-sectional design combined with MR analysis to explore the relationship between dietary antioxidant sources and NAFLD. The combination of both research methods provided more convincing results. However, our study still has some limitations that should be acknowledged. First information on the intake of exogenous antioxidants was obtained from the 24 h diet recall in the questionnaire, resulting in possible bias from the real situation. Second, owing to the absence of age- and sex-stratified GWAS data, we could not use MR analyses to validate the association between Vit E intake and NAFLD across sex and age found in cross-sectional studies. The subjects of the MR study were all Europeans, so we cannot be sure whether similar results can be obtained in other populations. In addition, MR analysis is a statistical method study and cannot elucidate the intrinsic pathogenesis, thus further relevant clinical and experimental validation is needed.

## Conclusion

Our research indicates a negative and linear relationship between daily vitamin E intake and NAFLD. Furthermore, Mendelian randomization results imply a connection between daily consumption of vitamin E and the occurrence of NAFLD. The recommended supplemental dose of vitamin E remains to be determined.

## Data Availability

Publicly available datasets were analyzed in this study. This data can be found here: NHANES.
