# Supplementary material for: Associations of dietary sources of antioxidant intake and NAFLD: NHANES 2017–2020 and Mendelian randomization
Source: Front Nutr. 2024 Oct 30;11:1447524. doi: 10.3389/fnut.2024.1447524 (PMC11565937; doi:10.3389/fnut.2024.1447524)

Supplementary Material

**Table S1. Genetic instrumental variables for Vitamin E**

| SNP | Effect allele | Other allele | Eaf | Beta | SE | *P* |
| --- | --- | --- | --- | --- | --- | --- |
| rs10926337 | T | G | 0.693534 | 0.00204269 | 0.000440556 | 3.54E-06 |
| rs114140227 | A | C | 0.982243 | -0.00708316 | 0.00157666 | 7.04E-06 |
| rs11577387 | A | G | 0.856637 | -0.00281279 | 0.000582763 | 1.39E-06 |
| rs117717573 | T | C | 0.978056 | 0.00613893 | 0.00137781 | 8.37E-06 |
| rs118073586 | A | C | 0.971581 | -0.00559314 | 0.00126569 | 9.92E-06 |
| rs12291674 | C | T | 0.97203 | -0.0055122 | 0.00123348 | 7.87E-06 |
| rs12552397 | G | A | 0.920728 | 0.00344141 | 0.000748938 | 4.33E-06 |
| rs1318526 | A | G | 0.808629 | -0.00230222 | 0.000517443 | 8.62E-06 |
| rs133932 | T | C | 0.98136 | -0.00681364 | 0.00149376 | 5.08E-06 |
| rs149751046 | G | A | 0.980099 | -0.00719505 | 0.00150632 | 1.78E-06 |
| rs16881098 | A | G | 0.900792 | 0.00301886 | 0.000676099 | 8.01E-06 |
| rs2058410 | C | G | 0.116559 | 0.00285511 | 0.000630629 | 5.97E-06 |
| rs2239302 | A | G | 0.943862 | -0.00411687 | 0.000902084 | 5.03E-06 |
| rs2753299 | G | A | 0.070088 | 0.00418446 | 0.000816583 | 2.99E-07 |
| rs2844803 | C | T | 0.509187 | -0.00187514 | 0.000404003 | 3.46E-06 |
| rs2972076 | G | A | 0.106967 | -0.0031421 | 0.000656246 | 1.69E-06 |
| rs35330354 | C | T | 0.981865 | -0.00718756 | 0.00159113 | 6.27E-06 |
| rs71431525 | G | A | 0.973563 | 0.00581413 | 0.00129958 | 7.68E-06 |
| rs74487803 | G | C | 0.979573 | -0.00632306 | 0.00143062 | 9.88E-06 |
| rs74623514 | G | T | 0.973099 | 0.00600047 | 0.0012952 | 3.61E-06 |
| rs74963516 | T | C | 0.950875 | -0.00457044 | 0.000954454 | 1.68E-06 |
| rs77646473 | T | A | 0.960553 | 0.00477997 | 0.0010764 | 8.97E-06 |
| rs9508621 | T | C | 0.62394 | -0.00190288 | 0.000421571 | 6.37E-06 |
| rs9983996 | G | A | 0.678798 | -0.00199577 | 0.000434302 | 4.32E-06 |

**Table S2. Mendelian randomization analysis of Vitamin E intake and NAFLD**

| Method | nsnp | Beta | SE | P | Lower CI | Upper CI | OR |
| --- | --- | --- | --- | --- | --- | --- | --- |
| Inverse variance weighted | 24 | -3.572934814 | 1.732789958 | 0.039212026 | -6.969 | -0.177 | 0.028 |
| MR Egger | 24 | -3.459974364 | 4.073702423 | 0.404838001 | -11.444 | 4.524 | 0.031 |
| Weighted median | 24 | -3.801629638 | 2.454681585 | 0.121447557 | -8.613 | 1.010 | 0.0223 |

**Figure S1. Results of leave-one-out sensitivity analysis for Vitamin E on NAFLD**


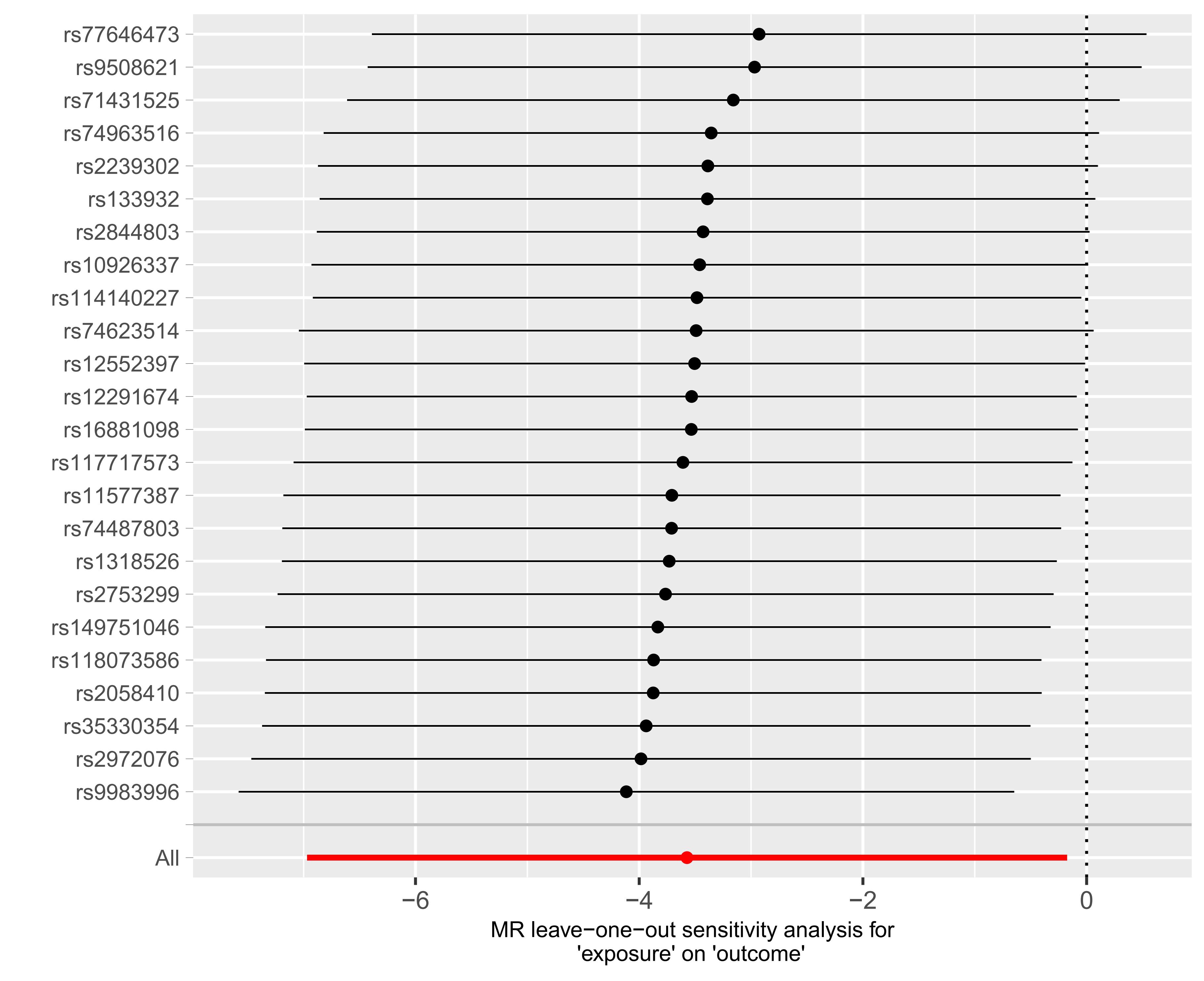

Supplement: Supplementary file 1 [file Table_1.DOCX]
